# Peptide-Lipid Interactions: Experiments and Applications

**DOI:** 10.3390/ijms140918758

**Published:** 2013-09-12

**Authors:** Stefania Galdiero, Annarita Falanga, Marco Cantisani, Mariateresa Vitiello, Giancarlo Morelli, Massimiliano Galdiero

**Affiliations:** 1 Department of Pharmacy, CIRPEB and DFM—University of Naples “Federico II”, Via Mezzocannone 16, 80134 Napoli, Italy; E-Mails: annaritafalanga@libero.it (A.F.); marco.cantisani@unina.it (M.C.); gmorelli@unina.it (G.M.); 2 Institute of Biostructures and Bioimages—CNR, Via Mezzocannone 16, 80134 Napoli, Italy; 3 Department of Experimental Medicine—Second University of Naples, Via De Crecchio 7, 80138 Napoli, Italy; E-Mail: mteresa.vitiello@unina2.it

**Keywords:** peptide, membrane, bacteria, virus

## Abstract

The interactions between peptides and lipids are of fundamental importance in the functioning of numerous membrane-mediated cellular processes including antimicrobial peptide action, hormone-receptor interactions, drug bioavailability across the blood-brain barrier and viral fusion processes. Moreover, a major goal of modern biotechnology is obtaining new potent pharmaceutical agents whose biological action is dependent on the binding of peptides to lipid-bilayers. Several issues need to be addressed such as secondary structure, orientation, oligomerization and localization inside the membrane. At the same time, the structural effects which the peptides cause on the lipid bilayer are important for the interactions and need to be elucidated. The structural characterization of membrane active peptides in membranes is a harsh experimental challenge. It is in fact accepted that no single experimental technique can give a complete structural picture of the interaction, but rather a combination of different techniques is necessary.

## 1. Introduction: Importance of Membrane Interacting Peptides

Peptide-membrane interactions are involved in numerous crucial biological processes, such as antimicrobial defense mechanisms, viral translocation, membrane fusion, functions of membrane proteins, transport of therapeutic compounds, disruption of integrity of membranes, and others. Membrane interacting peptides comprise a large family of diverse peptides exhibiting a broad range of biological activities and, therefore, continuously attract growing interest for their biomedical applications.

During peptide membrane interactions, both the peptide and the membrane may experience a series of structural changes. Thus, theoretical and experimental studies of peptide-membrane interactions constitute a challenging topic of research and complete understanding of the relationship between the structure of the peptide and the mechanism of interaction with lipids, as well as molecular details of this process, still remain elusive. However, it is of paramount importance to reveal the biological functions of membrane active peptides and to design peptides with tailored functionalities that may be exploited for therapeutic applications.

For example, antimicrobial peptides (AMPs) are able to recognize and kill many pathogens, and a number of these peptides have been identified as key components of the natural immune defense system. A related family of peptides, the so-called cell-penetrating peptides (CPPs), is capable of efficient translocation across the cell membrane, either by themselves or together with a molecular cargo, and are being explored as potential programmable drug delivery vectors [1]. There is no clear cut difference between AMPs and CPPs; in fact, some AMPs are able to cross membrane bilayers and some CPPs show antimicrobial activities, and thus, a clue to their different activities is derived from their interactions with the lipid bilayer. Other membrane-active peptides play a key role in cellular processes, such as membrane fusion, which is an ubiquitous process and represents a key stage in protein trafficking [2], exo and endocytosis [3], viral entry and exit [4,5]. The target of many of these sequences is the lipid bilayer itself, but some (such as peptide hormones [6–8] and bacterial toxins [9–12]) likely act on proteins located in the membranes and will not be the subject of this review.

Membrane interacting peptides can be classified according to their structure or their interaction with lipid membranes. They can assume different structures such as helical, β-stranded, mixed (containing both helices and strands) and cyclic, which are fundamental for modulating their membrane function. They usually contain amino acids with marked hydrophilic (Asn, Gln, Pro) or relatively hydrophilic (Phe, Trp, Tyr, Met) character, according to the Wimley and White hydrophobicity scale [13], which influences their position in membranes. The presence of positively (Arg, Lys, His) or negatively (Asp, Glu) charged residues is also a key feature, determining their interaction with target membranes.

Among membrane interacting peptides, those that cause membrane alteration or permeation are fundamental because they may be exploited for the obtainment of potential new antibiotics. On the contrary, peptides that do not disrupt the membrane modulate the structure and dynamics of the lipid bilayer and also play key roles in protein trafficking, exo and endocytosis. Intensive research efforts have been carried out to elucidate the interaction of peptides with lipid bilayers at atomic detail, taking into account the position, orientation, structure, and dynamics of the peptide in the lipid bilayer and its effects on surrounding lipids. Therefore, all this information is necessary to unveil their functioning modalities and to envisage their potential applications.

The complexity of the biological membrane is overwhelming and the multiple processes that occur simultaneously within this unique environment are just beginning to be understood.

A variety of structural models describing interactions between peptides and membranes have emerged and have been investigated in recent years, such as the transmembrane channel aggregates, the “barrel-stave” model, and the “carpet” mechanism [14]. The main differences between these models lie in the lipid structure around the pores and the pore stability. In the barrel-stave model, the lipids maintain a lamellar organization and the peptides form well-defined and stable bundles, which, when they are of a sufficient diameter, can serve as a pore. This is believed to be the arrangement of transmembrane helices in ion-conducting channels, either as part of a larger protein, or when organized through a self-assembly process. In the case of the toroidal-pore model, the lipids create a toroidal-shaped opening covered with the peptides in different orientations. Toroidal pores are generally less stable or transient compared to the barrel-stave pores [15]. It has been proposed that this latter mechanism is involved in the action of antimicrobial peptides, leading to cell leakage. In the carpet model, peptides accumulate on the membrane surface until its integrity is breached and transient holes are formed. These holes, when the peptides are in high concentrations, may result in the complete collapse of the membrane. Several peptide-membrane interaction mechanisms involve the insertion of the peptide into the membrane; in fact, during membrane fusion, fusion peptides (short hydrophobic segments of fusion proteins) destabilize the lipid bilayer structure by adopting an oblique orientation within the membrane. Other peptides, with a different distribution of hydrophobicity, adopt interfacial or transmembrane orientations relative to the membrane.

Many of such mechanisms include diverse stages of interaction between the peptides and the membrane or between various peptides, meaning that peptide-membrane interactions are complex and diverse phenomena. Depending on their composition, charge, and structure different peptides employ different interaction mechanisms with the membrane. In this review, we will focus on some of the most commonly seen scenarios in the studies of peptide-membrane interactions and on the different techniques that could be used to obtain deep details of the interactions.

## 2. Molecular Basis for Cell Selectivity: The Membrane Bilayer of Different Cells

Peptide membrane interactions are important for the selective targeting of peptides to specific types of cells. Biological membranes are complex structures composed mainly of proteins and lipids which do not form a single homogeneous mixture, being some regions are more enriched in some components of the mixture. The membrane bilayer of different organisms can be very different. For example, the plasma membrane of bacteria and mammalian cells differ in their composition and properties and this accounts for specific interactions of peptides with one or the other membrane. The cationic characteristics of AMPs mainly contribute to cell selectivity, because the surface of bacterial membranes is more negatively charged than that of mammalian cells. The cell membranes of bacteria are rich in acidic phospholipids, such as phosphatidylglycerol and cardiolipin. The cell walls also contain anionic molecules, such as lipopolysaccharides in the outer membrane of Gram-negative bacteria and teichoic acids and lipoteichoic acids in the peptidoglycan of Gram-positive bacteria. In mammalian cells, acidic phospholipids are usually only present in the inner leaflets of plasma membranes, while the outer leaflets are mainly composed of zwitterionic phosphatidylcholine and sphingomyelin, although negatively charged gangliosides can also be present as minor species.

Among the varied lipid components of mammalian membranes, the structure and physical properties of cholesterol (the main mammalian membrane active sterol) is rather different from that of other major membrane lipids; in fact, cholesterol is characterized by the presence of a hydrocarbon tail—a ring structure region with four hydrocarbon rings—and a hydroxyl group, and in lipid membranes, is slightly tilted with respect to the bilayer normal configuration, with the hydroxyl group positioned in the lipid head group region. This feature induces very different energies of interaction between proteins and cholesterol compared to other membrane lipids. In particular, depending on the temperature, cholesterol has different effects on membrane fluidity. At high temperature, it interferes with the movement of phospholipid fatty acid chains, making the membrane less fluid while at lower temperatures it prevents membranes from freezing and maintains membrane fluidity. These characteristics determine a segregation of components in membranes with the formation of cholesterol enriched microdomains (lipid rafts) [16]. The factors that facilitate the interaction of proteins with cholesterol are varied and are not yet completely understood; but it is widely accepted that the presence of membrane-stabilizing cholesterol in mammalian cells protects the cells from attack by AMPs.

## 3. Examples of Membrane-Interacting Peptides: AMP, CPP, Viral Peptides, Amyloidogenic Peptides

### 3.1. Antimicrobial Peptides

AMPs were first discovered some decades ago in plants, insects, amphibian venoms and tissue extracts [17]. Numerous antimicrobial peptides have been isolated from natural sources (such as temporins, gomesins, defensins [18–20]) and others have been *de novo* designed and synthetically produced [21–23]. An online database of AMPs can be found at http://www.bbcm.univ.trieste.it/*tossi/amsdb.html or at http://aps.unmc.edu/AP/main.php. AMPs have the ability to kill pathogenic microorganisms, including Gram-positive and Gram-negative bacteria, viruses, protozoa, and fungi; thus providing a first unspecific defense mechanism against microbial invasion. Moreover, they play an important role in the innate immune system of higher organisms (plants, insects, amphibians, and mammals) [24,25], which produce them on epithelial surfaces or directly in endothelial and phagocytic cells, thus exhibiting a defense system for prevention of colonization and infection.

AMPs generally consist of less than 60 amino acid residues, and bear a positive net charge, which is often due to the Lys and Arg residues [26–31]. Additionally, they often consist of nearly 50% hydrophobic residues. Furthermore, in their folded state, these peptides exhibit spatially separated hydrophobic and charged regions and show amphipathic properties [32], a feature which is typical of membrane active sequences. A unique interplay between the initial electrostatic interaction and the subsequent hydrophobic partitioning confers AMPs the ability to be highly water soluble, but yet able to interact strongly with phospholipid bilayers. Despite these similarities, their structure is very diverse, and they vary considerably in length, amino acid sequence, and type of secondary structure, rendering their classification extremely complex.

Considering their secondary structure, AMPs can be divided into four different groups [33]: (i) linear, α-helical peptides without the presence of cysteines in their sequence, such as melittin; (ii) β-sheet peptides that contain two or more disulfide bridges in their peptide structures, such as defensins; (iii) peptides with intermolecular disulfide bonds, exhibiting loop/hairpin-like structures, such as bactenecin; (iv) peptides with predominance of one or more distinct amino acids, such as the proline/arginine-rich peptide Bac7.

AMPs display antimicrobial activity at micromolar concentrations or below. Besides membrane disruption, their bactericidal mode of action may involve interference with metabolic processes or targeting of cytoplasmic components [34]. Predominantly, they act by disrupting the integrity of cell membranes, through interaction of their cationic domains with negatively charged cell surface components, mostly phospholipids. It is also known that at least some of these peptides can not only act directly as microbe killers but also play an important role in tissue processes. The defensins, for example, are described to be involved in various signaling events, such as wound repair, cell migration or chemotaxis [21,22,35].

As a consequence of their membrane target and mechanism of action, development of bacterial resistance towards AMPs is much less likely to happen compared to conventional antibiotics. Therefore, AMPs have been brought into focus as potential candidates for a new generation of antibiotics.

### 3.2. Cell-Penetrating Peptides

Some intracellular proteins are able to pass through the cell membrane. Since the earliest discovery of this property for the HIV Trans-Activator of Transcription (Tat) and *Drosophila melanogaster* Antennapedia proteins [36,37], many other proteins that contain peptide domains responsible for membrane-translocating properties have been identified.

A wide range of different applications of CPPs has been described, including transport of proteins, oligonucleotides, quantum dots, polysaccharides, nanoparticles, chemotherapeutics, polymers, and liposomes [38–47].

Like AMPs, CPPs carry a positive net charge that is due to a large amount of basic amino acids (such as Arg and Lys) in their peptide sequence, and are also characterized by an amphipathic structure. The growing number of CPPs discovered so far is divided into natural, synthetic and chimeric peptides [48]. The mechanism of cellular uptake of CPPs is still controversial and although it has been the subject of many studies, a unifying pathway remains elusive [49]. In particular, both endocytotic and nonendocytotic routes have been proposed and, depending on factors such as the peptide concentration, the cell line, the cargo, and the overall incubation conditions, it has been speculated that often more than one uptake pathway is possible [49–51].

CPPs are overall hydrophobic, an essential characteristic for the interaction with the lipid part of the cell-membrane. The combination of a charged and a hydrophobic domain plays a key role in the interaction with natural membranes; the charged moieties are involved in the initial interaction with the bilayer while the hydrophobic domain is crucial for insertion.

Most CPPs are amphipathic, consisting of two domains: a hydrophilic (polar) domain and a hydrophobic (non-polar) domain. Such an amphipathicity can occur at the levels of their primary structure or secondary structure. Primary amphipathic peptides correspond to the sequential assembly of a hydrophobic domain with a hydrophilic domain separated by a spacer domain; while secondary amphipathic peptides are generated by the conformational state which positions hydrophobic and hydrophilic residues on opposite sides of the molecule.

### 3.3. Viral Peptides

All enveloped viruses share common steps of entry to the host mammalian cell using two main routes, either by the endocytic or nonendocytic pathways. Clathrin coated vesicles, macropinocytosis and caveolae are all pathways exploited by viruses preferring the endocytic route, while the non-endocytic pathway involves direct crossing of the plasma membrane at neutral pH. Regardless of the chosen route, the basic mode of entry by enveloped viruses is through membrane fusion, an essential and ubiquitous mechanism in most cellular events. Fusion is mediated by viral membrane proteins which undergo remarkable conformational modifications as a consequence of a trigger that is represented either by low endosomal pH or receptor binding. These conformational changes lead to the exposure of hydrophobic peptides, loops or patches (the so-called fusion peptides), which then interact with and destabilize one or both of the opposing membranes. Three different classes of viral fusion proteins have been identified to date based on their common post-fusion structural motifs [52]. These are: (i) class I fusion proteins, characterized by trimers of hairpins containing a central α-helical coiled-coil structure; (ii) class II fusion proteins, characterized by trimers of hairpins composed of β structures; (iii) class III fusion proteins that show features of both classes.

However, the overall activity of the three classes of fusion proteins, regardless of their structural and biochemical differences, seems to induce membrane fusion in a similar manner. In fact, after fusion activation all fusion proteins will end up forming a similar hairpin structure. The principal element of the fusion machinery is always represented by a fusion peptide able to be inserted into cell membranes and drive membrane destabilization. Following activation by receptor binding or acidification of the endosomal compartment, the fusion peptide is projected toward the most external side of the glycoprotein where it is inserted into the target membrane. The structural changes in the fusion protein end up in the correct position of the target membrane, held by the peptide or fusion loops, and the viral membrane, held by the trans-membrane region of the fusion protein. Further remarkable refolding steps result in the merging of the two lipid layers and the consequent release of the viral nucleocapsid inside the host cells.

The precise molecular events that lead to fusion are still unknown. Surely, the membrane leaflets have to be modified in order to allow accomplishment of fusion. It is now widely accepted that fusion peptides are capable of bilayer destabilization, by a combination of lipid head group charge neutralization and a deep localization of the peptide in the bilayer [5,53]. It is believed that a key role is played by the spatial distribution of hydrophobic and hydrophilic residues, which determines their oblique insertion and constitutes the initial step in membrane fusion [54,55].

In contrast, non-enveloped viruses, which by definition lack an outer membrane, mediate membrane fusion via utilization of previously sequestered viral lytic factors. The process of non-enveloped virus’ entry generally starts with the interaction with a cellular stimulus (e.g., receptors, low pH, proteases) at the penetration site which drives a conformational change that triggers the release of viral components with membrane lytic activity. This is followed by the binding of the small viral peptides or their hydrophobic domains with membranes to allow the transfer of viral genetic material and/or nucleocapsid to the cytoplasm. The molecular details of membrane disruption and the structural characteristics of the domains involved vary for different viruses. Examples of membrane altering sequences derived from several nonenveloped virus capsid proteins are: (i) peptides generated by autocatalytic cleavage of a precursor such as in the case of the γ peptide of nodaviruses [56]; the VP4 and the *N*-terminal region of VP1 of picornaviruses [57]; and the μ1N of reoviruses [58]; (ii) peptides generated by trypsin cleavage such as in the case of VP4 of rotavirus [59]; (iii) peptides generated by cellular proteolysis such as the protein VI of adenoviruses [60]; (iv) peptides generated by viral proteolysis such as pep46 and additional peptides of birnavirus [61,62].

Notwithstanding their origin, viral entry peptides are recently finding applications for the design of novel drug delivery tools due to their ability to cross membrane bilayers [1,63,64].

### 3.4. Amyloidogenic Peptides

Amyloids are insoluble fibrillar aggregates of proteins/peptides [65]. Accumulation of these aggregates in organs is often associated with a large variety of human diseases such as Alzheimer’s, Parkinson’s and Huntington’s disease. The mechanism of formation and the possible role of amyloid aggregates are still unclear. Amyloids have cross-β-sheet quaternary structures with β-strands aligning perpendicular to the axis of the fibril and these β-strands are either parallel or anti-parallel [66]. Though, the detailed mechanisms of cytotoxicity are poorly understood, literature data support the hypothesis that these deposits might disrupt the architecture and integrity of cell membranes, resulting in death of the cells [67]. Identification of nucleating residues and of residues responsible for this oligomeric tendency could improve understanding of structure and function relationships as well as the molecular mechanism of folding and aggregation. A combination of biological, chemical or physical approaches is necessary for the unravelling of the aggregation mechanism and for the development of successful anti-amyloidogenic agents to control aggregation [68].

Amyloidogenic peptides are natively unfolded peptides which interact with lipid membranes and are involved in the formation of amyloid fibrils [69]. The formation of globular aggregates due to interactions of these peptides with lipids is the basis for the complex aggregation process correlated with all amyloid-related diseases [70]. The structure of the amyloidogenic peptides may change from random coil to α-helix or β-sheet and in particular, membrane interaction has been demonstrated to involve a conformational rearrangement from α-helical to β-sheet [71–74]. Amyloid aggregation is a multistep process in which peptides initially cluster together into soluble dimers, trimers or other oligomers whose shape and composition is not well characterized, followed by interaction with lipids and causing cellular toxicity; to make this scene more complicated, the islet amyloid polypeptide has been demonstrated to insert into phospholipid monolayers most likely as monomer [75]. A disruption of the membrane integrity seems to be the most likely explanation for toxicity. In fact, fibril growth causes leakage due to the fibrils grown on the lipid surface, which penetrate the membrane structure. Fully grown and stable fibrils are released from the vesicle surface. Oligomers may disrupt the vesicle membrane by adsorption to its surface where they interfere with the packing of lipids or by growing into fibrils.

Most of these peptides have been demonstrated to be capable of channel formation in lipid bilayers and it has been proposed that this may represent one of the pathogenic mechanisms. The channels formed exhibit a number of common properties, including irreversible, spontaneous insertion into membranes, production of large, heterogeneous single-channel conductances, relatively poor ion selectivity, inhibition of channel formation by Congo red and related dyes and blockage of inserted channels by zinc. There are also other possible mechanisms for membrane permeabilization which may involve interactions with specific membrane receptors [76] or distortions in the phospholipid bilayer packing, causing membrane instability which occurs without any evidence of channel or pore formation [77]. *In vivo* amyloid peptides have been shown to disrupt intracellular calcium regulation, plasma membrane potential, mitochondrial membrane potential and function and long-term potentiation in neurons. The formation of the β-sheet conformation from native protein structures can be induced by high protein concentrations, metal binding, pH, amino acid mutation and interaction with lipid membranes [78–80]. Pore formation appears to be a spontaneous process and evidences suggest that several steps are critical. First, destabilization of the native structure and formation of the β-sheet conformation must occur, in aqueous solution or facilitated by contact with lipid membranes. Oligomerization of the amyloid protein is then mediated by the β-strands. Several hypotheses have been proposed which point toward amyloid oligomers of intermediate size as the main cytotoxic species of amyloidogenic peptides [77]. Recently, it has also been proposed that toxicity is not linked to specific prefibrillar aggregates but rather to the ability of these species to grow and undergo fibril formation, which depends on the presence of monomeric amyloidogenic peptide [81,82]. Insertion of the oligomer appears to take place spontaneously, although there may be a contribution of pH and/or membrane potential. Very little is known about the structure of amyloid pores, but given that the amyloid peptides must acquire β-sheet conformation to aggregate and polymerize, it has been hypothesized that amyloid pores may in fact be β-sheet barrels similar to the pores formed by alpha-latrotoxin, Staphylococcal α-hemolysin, anthrax toxin and Clostridial perfringolysin and, in general, β-barrel membrane proteins [11,12,83].

## 4. Experimental and Theoretical Techniques

**Figure 1:**
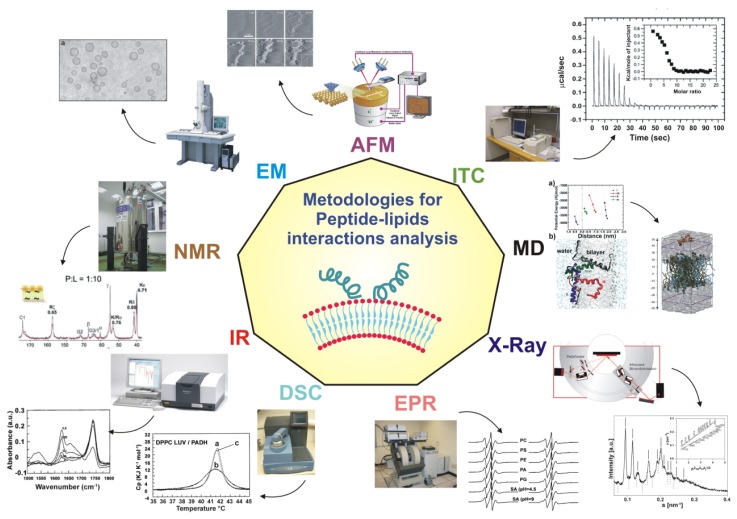
Schematic representation of the most recent and significant methodologies which are usually combined in biophysical studies of membrane interacting peptides providing complementary information. The list of reference is not exhaustive, a few examples are provided IR: Infrared Spectroscopy; NMR: Nuclear Magnetic Resonance; EM: Electron Microscopy; AFM: Atomic Force Microscopy; ITC: Isothermal Calorimetry; MD: Molecular Dynamics; X-ray: X-ray Crystallography; EPR: Electron Paramagnetic Resonance Spectroscopy.

The binding, location and orientation of a peptide relative to a lipid bilayer as well as lipid rearrangements in the presence of peptides are the most important features of peptide-lipid interactions. During the last decades, several experimental techniques have been developed and applied to biological systems, which differ in the nature and size of the sample, the sensitivity and the type and resolution of the information that they can provide. This section describes some of the most recent and significant methodologies which are usually combined in biophysical studies of membrane interacting peptides, since they provide complementary information (Figure 1).

There are techniques that describe the morphological changes induced by peptides such as electron microscopy (EM), atomic force microscopy (AFM) and fluorescence imaging [84–87]. EM and AFM have a great spatial resolution but the sample preparation may generate artifacts, whereas classical fluorescence has a large sensitivity but uses dyes that may perturb the system, and has a limited resolution. There are other techniques such as calorimetry, which allows identification of changes in the thermodynamic properties of the membrane [88] solid-state nuclear magnetic resonance (NMR) and X-rays, which describe global phase changes [88–90].

Techniques such as fluorescence, electron paramagnetic resonance spectroscopy (EPR), infrared spectroscopy (IR), circular dichroism (CD), surface plasmon resonance (SPR), X-rays and NMR illustrate interaction and are capable of describing both peptide and lipid structure and dynamics. The secondary peptide structure upon binding, degree of penetration and orientation can easily be obtained from IR, CD and EPR studies [91–103]. NMR is the only technique capable of yielding both the topology and the three-dimensional structure of peptides in membranes [104–107].

Computational methods have been widely used for studying peptide membrane interactions. Computer simulations are in fact an essential tool in the study of dynamic processes in biology and chemistry, providing data on sizes and time scales that are not accessible to experimental techniques. The mode of insertion of helical peptides has been predicted with success [108,109], but this approach is limited by the difficulties associated with the presence of peptide-peptide interactions and the fact that gradients of mobility and dielectric constant across the bilayer are not accounted for. In spite of this large collection of experimental techniques, it is still difficult to obtain complete and detailed information of the actual modes of peptide-membrane interactions. Furthermore, to interpret the obtained data, a model is required, and its construction may be a challenge in itself. A description of the interaction mechanism can be only derived using several complementary experimental techniques.

### 4.1. Structural Studies: X-Ray, NMR, EPR, AFM, NR

Experimental techniques that have been used to get structural information include X-ray diffraction, Neutron Reflectivity methods (NR) as well as NMR, EPR, AFM.

The possibilities of using X-ray diffraction to gain detailed insights into peptide-membrane systems are limited, because of the lack of long-range crystalline order. Crystal structures have been obtained only of a few membrane-bound peptides, which were crystallized from water or co-crystallized using organic solvents as membrane mimetics [20,110].

Neutron reflectivity (NR) is a well-established technique for studying membranes at the solid/water interface, and provides detailed information on the structure and on the composition of the interface, but also of buried materials. Because of the large penetration depth of neutrons, due to their weak interactions with almost any material, *in situ* measurements at solid-liquid interfaces can be performed. An important advantage of this method is the absence of sample damage, as frequently observed in X-ray reflectivity experiments, even upon prolonged exposure to the neutron beam. The information that can be extracted from a single neutron reflectometry experiment includes the film thickness, the scattering length density profile across the film and the surface roughness σ at the solid-liquid interface. Several applications to the study of membrane interacting peptides have been reported recently [111,112].

NMR—and specifically, solid state NMR—has numerous applications in peptide membrane interaction studies; in fact, a series of NMR experiments have provided important insights about peptide orientation [74,107,113–117]. NMR is a unique tool that uses samples that are solid or liquid, viscous or fluid, and can give access to very accurate local distances, orientations or dynamics and also the full high resolution three-dimensional structures by measuring 2D or 3D spectra; besides protons, NMR exploits ^13^C, ^15^N and ^2^H [118]. A variety of membrane mimetics are available for NMR studies which can be selected according to the experiment to be performed. In particular, the investigation of membrane bound peptides is actually performed in membrane mimetic or model membrane systems such as micelles, bicelles, small unilamellar vesicles (SUVs) or multilamellar vesicles [107,118–121]. SDS is a negatively charged detergent that has been used as a model for bacterial membranes, but it can influence the secondary structure inducing conformations that are not found in other membrane mimetics. DPC (dodecyl-phosphocholine) or DHPC (dihexanoylphosphatidylcholine) structurally resemble the components of most eukaryotic biological membranes and can preserve the three-dimensional structure of bound peptides as well as their activity [107,118,122–124]. Micelles and SUVs are used because of their small sizes, but they possess a large surface curvature, which might in some cases lead to structures or membrane topologies different from those found in larger vesicles or real membranes. One solution NMR approach to obtain information about the localization of peptides and proteins bound to micelles is the measurement of the Nuclear Overhauser Effect (NOE) which was used to confirm the topology of the antimicrobial hexapeptide Cyclo (RRWWRF) bound to SDS and DPC micelles [125]. Another solution NMR-based approach for obtaining information within membrane-mimetics uses residual dipolar couplings (RDCs) [126]. The RDC size depends on the nature, distance and angle of an internuclear vector such as the ^1^H-^15^N bond relative to a molecular reference frame. The relative orientations of α-helices in the membrane can be determined, as well as any deviation of helices from their ideal geometry, like bends or kinks; the only information that cannot be obtained is the immersion depth. Another of the most frequently used solution NMR methods for investigating the orientation and localization of peptides bound to membrane-mimetics uses the effect of paramagnetic probes on the peptide itself or tags on the peptide [127]. For example, through the introduction of 5-, 12- or 16-doxylstearate to dodecylphosphocholine micelles, the interior of the micelles is made paramagnetic and it is possible to obtain a qualitative picture about the peptide orientation. A more quantitative approach to obtain the orientation and location inside a micelle is the use of the depth dependent partitioning of oxygen towards hydrophobic environments [128]. The introduction of large reporter groups to peptides might help in understanding their interaction with the membrane but in some cases affects their structural and functional properties. It is also possible to add a paramagnetic compound to the solution surrounding the micelle. In this case, the relaxation enhancement affects spins close to the surface of the micelle [129]. In solution NMR spectroscopy, anisotropic interactions of nuclei with the magnetic field are averaged to their isotropic values by rapid molecular reorientation; thus, for the dipole-dipole coupling, chemical shift anisotropy and quadrupolar coupling interactions, the isotropic values are zero, and consequently, these interactions cannot be observed in solution NMR spectroscopy. In solid-state NMR spectroscopy, the anisotropic interactions result in severely broadened lines, thus impeding the resolution of signals from different sites due to signal overlap in spectra of polypeptides [130]. Two different approaches for overcoming this problem are used. The first is MAS (Magic Angle Spinning) where the anisotropic interactions are averaged by fast spinning of the sample around the “magic angle”; the obtained spectra are very similar to those obtained by solution NMR spectroscopy and information on molecule orientation relative to the external magnetic field is lost, but can be regained either by analysis of the spinning sidebands, or by the recoupling of weak dipolar couplings using rotor-synchronized pulses. The second approach relies on the use of bilayers, which are uniaxially oriented with respect to the magnetic field; this approach results in a single resonance line from each isotopically labeled site in the polypeptide, while still retaining the orientational information contained in the anisotropic interactions.

EPR has been widely used to monitor membrane interactions [99,131–134]. One approach exploits the anisotropic nitroxide spin label 2,2,6,6-tetra-methylpiperidine-1-oxyl-4-amino-4-carboxylic acid (TOAC) [99,133,135–138]. TOAC is introduced by chemical synthesis into the studied peptide. Compared to NMR, EPR has the considerable advantage of being applicable to much smaller amounts of peptide due to the intrinsically higher sensitivity of the technique, however, the peptides have to be chemically modified which is laborious and may influence its behavior in the hydrophobic environment.

AFM has also been used for the characterization of peptide-membrane systems [84–87]. Destabilization of a bilayer due to fusion peptides, and restructuring of the membrane in the presence of specific peptides are just some of the examples where AFM has been used to get information about peptide-membrane interactions. Despite the level of the in-depth structural characteristics captured by AFM, the method generally does not provide any chemical information of the system under study. Recent applications of AFM to membrane active peptides validate the applicability of a combined AFM and fluorescence approach to provide a clear and detailed description concerning the mode of action of membrane-active molecules [84].

### 4.2. Computer Simulations

The understanding of the interaction of peptides with lipid bilayers at atomic level requires characterization of the position, orientation, structure, but also dynamics of the peptide in the lipid bilayer and its effects on surrounding lipids. Molecular dynamics (MD) simulation is a powerful research tool to theoretically study peptide-membrane interactions, which can provide a detailed description of these processes at a molecular level. However, considerable obstacles need to be faced to obtain accurate results, due to inexact force fields and other approximations in simulation methodology, and to the relaxation times of these phenomena, which are typically longer than those presently accessible to MD. In fact, in order to fully describe peptide-membrane interactions, it would be necessary to consider the behavior of an individual or several peptide molecules, to elucidate the dynamics of the peptide-membrane assembly process, to provide a link between the observed behavior and the data measured in experiments and to systematically explore a large number of systems.

Over the years, a number of theoretical and computer simulation approaches have been developed to describe membrane behavior and peptide-membrane interactions, which vary in the way the peptide-membrane system is modeled, and thus, in the type of information that can be obtained from each particular model [108,139–143].

Several atomistic molecular simulation studies attempted to address long scale peptide-membrane interactions in their full complexity showing pore formation [15,144], peptide translocation [145] or micropinocytosis [146]. A main limitation of these investigations is the definition of the correct initial conditions, which added to the intrinsic time limits, and conditions the reliability of the mechanisms and observed final states. In fact, none of these simulations spanned a timescale beyond several hundreds of nanoseconds.

These limitations in time and length scales led to the development of coarse-grained approaches (CG) for the study of complex biomolecular phenomena, in which the time problem may be circumvented at loss of resolution [147]. CG approaches are based on the idea of systematically reducing the level of detail in the way the system is represented, and thus, increasing the time/length scale of the simulation.

Recently, the CG models have been further extended, representing each amino acid according to its properties, such as tendency to form hydrogen bonds, hydrophobicity/hydrophilicity and charge, or representing the backbone of the amino acid and the side chains as different beads [148–152]. Moreover, newer force fields have been optimized to reproduce some key properties of amino acids, such as oil/water partition coefficients and association constants between different amino acids, including the effect of temperature and membrane composition on the properties of liposomes [153]. The model has been shown to accurately describe peptide-membrane interactions for several helical peptides and to illustrate a possible mechanism for the formation of a toroidal pore by magainin-H2 [144] as well as to describe the self-assembly of cyclic peptides near or within membranes and the formation of a barrel-stave pore by LS3 synthetic peptide [154].

### 4.3. Other Experimental Approaches to Understand Peptide Location in the Bilayer: SPR, Fluorescence, Calorimetry, CD

There are also other experimental techniques that can be used in order to map the position of different molecules in a peptide-lipid bilayer system.

Fluorescence spectroscopy is widely used for determining the approximate position and orientation of lipid associated peptides and membrane affinities. Both the peptide and/or the surrounding lipids could be labeled with a fluorescent tag. This technique also allows investigation of the fast kinetics of peptide insertion into the membrane. Membrane bound peptides often contain, or are modified to contain, a tryptophan (Trp) residue which possesses intrinsic fluorescence. Moreover, Trp is extremely sensitive to the polarity of its surrounding and can be used for calculating binding affinities for membrane-mimetics. Upon interaction with a hydrophobic environment, the Trp fluorescence emission is shifted to shorter wavelengths (blue shift) and decreases in intensity [101,155,156]. The degree of blue shift can be correlated with the membrane insertion depth. Additional information may be obtained using either aqueous or membrane-embedded quenchers. Typical aqueous quenchers are iodide ions or acrylamide, while brominated or methyl-coumarin labeled phosphocholines can be used to quench the fluorescence in the hydrophobic environment [157,158]. Several experiments can be used to probe the effect of peptide interaction with the membrane, such as membrane fusion, leakage or inner monolayer fusion [94,96,98,159–162].

Surface plasmon resonance (SPR) has become one of the most important techniques for studying macromolecular interactions. Recent advances in the preparation of stable membrane-like surfaces and the commercialization of sensor chips have enabled widespread use of SPR in the study of membrane interactions. The number of published papers has increased steadily in the last few years, especially since Biacore introduced the HPA and L1 chips, dedicated to lipid systems [100,163–169]. The HPA sensor chip contains hydrophobic alkanethiol chains, which are covalently bound to its gold surface, and a lipid heteromonolayer is created by adding liposomes to the chip; the complete coverage of the surface with a polar lipid monolayer generates a membrane-like environment where analytes in aqueous buffer interact with a lipid monolayer [170]. The L1 sensor chip contains hydrophobic alkanethiol chains, with exposed polar head groups, and a lipid bilayer is created by adding liposomes to the chip.

**Figure 2:**
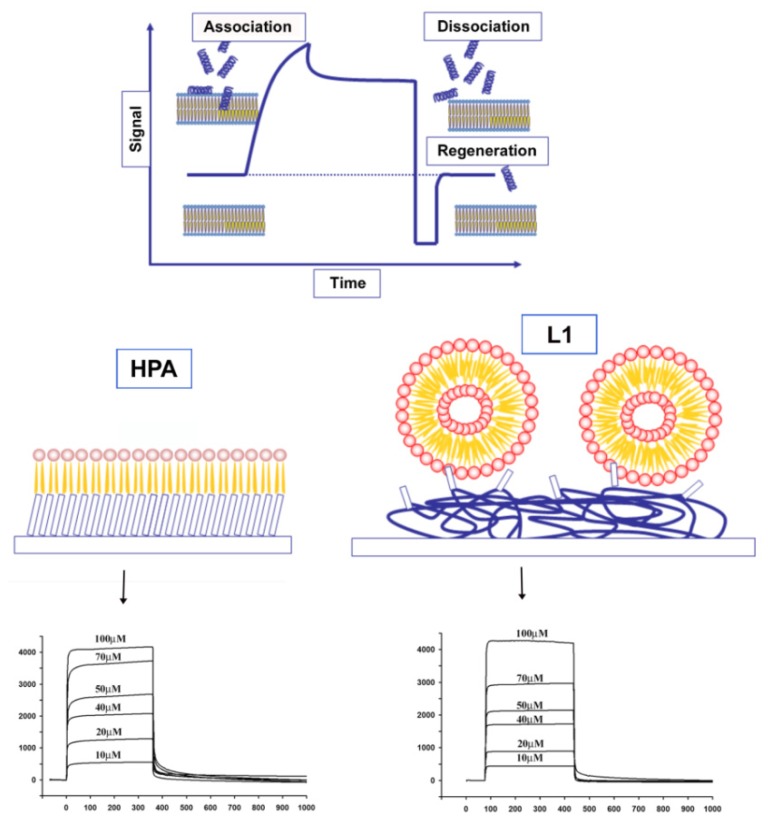
Surface Plasmon Resonance (SPR) and use of different chips for the study of peptide membrane interactions: L1 and HPA. Due to structural differences between the two chips, it is possible to distinguish between surface adsorption in the HPA chip and insertion into the hydrophobic core of the membrane in the L1 chip.

SPR studies, in general, provide both qualitative and quantitative data on molecular interactions (Figure 2).

The typical qualitative study allows the determination of lipid specificity of a membrane-binding peptide, which very often is the key regulatory step of peptide action. Lipid specificity can be easily studied by manipulating the lipid composition of the immobilized membrane. The visual inspection of binding curves can immediately deliver qualitative information to describe how peptides bind to some kinds of lipids. Moreover, it is possible to determine the apparent rate and affinity constants from sensorgrams, especially when the differences between various conditions, either different types of membranes/buffers/pH or different variants of systems studied, are subtle. The affinity constants can be directly determined from the equilibrium binding responses over a range of peptide concentrations, by fitting the data to a Langmuir adsorption isotherm. In addition, binding constants can be determined directly from the sensorgrams by numerical integration analysis [171–173]. This is conveniently done by Biacore BIAevaluation software or other dedicated programs, through the use of appropriate binding models. Usually, a significantly improved fit is obtained for membrane interacting peptides when using the two-state reaction, suggesting that there is likely to be at least two steps involved in the interaction between the peptide and hybrid bilayer membrane surface [14,137]. A first step corresponds to the actual binding of the peptide to the surface, and a second step corresponds to the insertion of the peptide into the hydrophobic core of the membrane.

Additional information about the mechanism of membrane association can be obtained when both HPA and L1 chips are used. Due to structural differences between the two chips, it is possible to distinguish between surface adsorption in the HPA chip and insertion into the hydrophobic core of the membrane in the L1 chip. If the peptide binds only to the water–lipid interface, and binding is not accompanied with deeper penetration, then similar equilibrium constants can be observed with both chips. On the other hand, if the peptide inserts deeper in the membrane and needs a transmembrane compartment, then no binding is observed in HPA. A difference between the affinities of the peptides to lipid monolayers, compared to bilayers, indicates the contribution of the membrane hydrophobic core to the binding process. The ratio *K*_Abilayer_/*K*_Amonolayer_ provides an indication of the depth of penetration into the membrane core. A ratio of ~1 indicates that the peptide is surface localized, and a ratio above ~10 indicates that the peptide has been inserted into the lipid bilayer. Several studies have reported this behavior, using calculations of different binding affinities of peptides on L1 or HPA chips [168–174]. In summary, the use of SPR measurements on various lipid systems enables differentiation between distinct steps during the mechanism of action of membrane-active peptides.

Isothermal calorimetry (ITC) is another methodology which has proved to be very useful for the study of peptide membrane interactions. The development of high sensitivity titration calorimeters has allowed the measurements of binding reactions with solutions in the micromolar concentration range. A complete thermodynamic picture can be obtained to provide information about the driving forces of the binding reaction. ITC can also be used to investigate secondary processes accompanying peptide-membrane interactions, such as membrane permeabilization, peptide induced lipid phase transitions, peptide aggregation at the membrane surface and peptide conformational changes [19].

The phase transition temperature and thermodynamics of lipid phase transitions are extremely sensitive to the presence of exogenously added compounds. Differential scanning calorimetry (DSC) has been used in a variety of peptide lipid studies to monitor changes in these parameters and obtain valuable information regarding the ability of peptides to interact and/or disrupt the lipid acyl chain packing also providing insight into their interaction mechanism [88,175,176].

CD spectroscopy is used to study the secondary structure of peptides and its changes in different environments such as temperature, pH, ligands, or denaturants [177]. The orientation of α-helical peptides in a multilayer lipid membrane can be determined using light transmitted perpendicular and under oblique angles relative to the membrane plane [178,179]. For example, this method has been used to determine the orientation of the α-helical peptide alamethicin in diphytanoylphosphatidylcholine multilayer [179], to determine the effect of the cationic antimicrobial peptide novicidin on membrane integrity [180] or to study the effect of membrane composition on the orientation of the antimicrobial peptides aurein 2.2 and 2.3 in mixed phosphatidylglycerol/phosphocholine membranes [181].

## 5. Examples of Applications: Antimicrobial Compound, Drug Delivery, Antiamyloidonegic Compound

**Figure 3:**
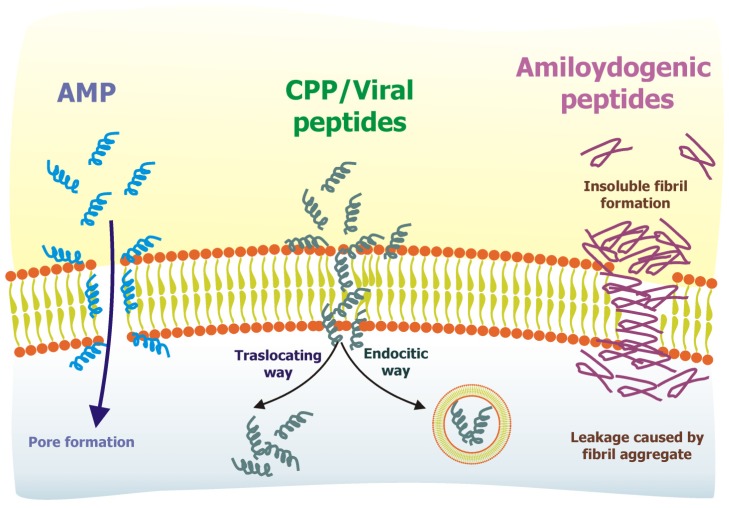
Mechanism of action of membrane-active peptides. Depending on their composition, charge and structure, different peptides employ different interaction mechanisms with the membrane. Examples of different membranotropic peptides: AMP (antimicrobial peptides); CPP (cell penetrating peptides), viral peptides and amiloydogenic peptides. Both CPP and viral derived peptides are able to penetrate into the cell through endocytosis or direct translocation. CPP have been mainly described to enter by endocytosis, while viral peptides seem to prefer a direct translocation mechanism.

The interaction of peptides with membranes is a topic of great interest, both for the purpose of understanding the mechanism of action of membrane-active peptides as well as for designing peptides which can modulate membrane properties and may have applications for human health (Figure 3).

The great interest in biophysical studies of the interaction between AMP and lipids is in part due to the rapid emergence of antibiotic-resistant bacterial strains. The hope is that the understanding of the molecular mechanisms used by AMPs for membrane perturbation may allow the design of novel peptide antibiotics which could be used as an alternative or in combination with conventional antibiotics. The biological activity of AMPs stems from their ability to perturb the lipid bilayer structure of membranes. Moreover, some of this peptides exhibit high specificity towards their target membrane and are toxic to humans while others are toxic only to microorganisms. The understanding of the damaging properties on specific target membranes plays a key role in the rationale design of novel peptide antibiotics, and is thus pharmaceutically relevant.

The transport of drugs or drug delivery systems across the cell membrane is another complex biological process, often difficult to measure because of its dynamic nature. Biophysical investigations play a critical role in understanding the mechanism of cellular uptake, the toxicity of drugs and to optimize drug delivery systems [182]. Model membranes offer several advantages over the use of live cells in that: (1) experiments can be performed in conditions that cells may not withstand; (2) the mechanism of transport can be more easily deduced; (3) the interactions of drugs and drug-delivery systems with biological membranes can be predicted. Model membranes mimic many aspects of cell-membrane lipids and have been very useful in helping investigators to discern the roles of lipids in cellular interactions. One can use drug-lipid interactions to predict pharmacokinetic properties of drugs, such as their transport, biodistribution, accumulation and hence efficacy, and therefore could help to study the mechanisms of transport, based on the structure and hydrophilicity/hydrophobicity of drug molecules. These studies can be used to design and develop efficient drug delivery systems [48,183,184]. Changes in the lipid composition of cells and tissue in certain disease conditions may alter biophysical interactions, which could be explored with model membranes.

Membrane translocation is a necessary step for membrane permeabilization, and in fact, CPPs and AMPs are very similar molecules, and although being treated differently, both interact with the bilayer. At high concentration, peptides reported as CPPs perturb the membrane and become membrane permeabilisers, whereas at low concentrations, AMPs may reach cytoplasmic targets before membrane permeabilization. Cellular membranes of target cells where the activity of these two classes of peptides are evaluated are quite different. In fact, CPPs are evaluated against mammalian cells, whereas the target of AMPs is the bacterial cell. The bacterial membrane has a higher percentage of negatively charged lipids and does not contain sterols, thus, different effects reported with CPPs and AMPs can arise from the differences in membrane composition, and other factors which modulate peptide affinity and membrane destabilization. Another class of membranotropic peptides that is an object of active research for the design of novel delivery tools is represented by viral-derived peptides.

The membrane affinity and the capacity to destabilize the bilayer dictate the extent to which a peptide enters the cell by a physical mechanism at the expense of the endosomal pathway. Most CPPs induce an endocytotic mechanism with the first step of their cellular entry being the electrostatic interaction with the cell surface. In particular, conformational changes modulate peptide/lipid interactions and the strength of these interactions might determine whether the peptides remain entrapped at the plasma membrane or not and whether they follow an endocytotic route or induce membrane disorganization with direct translocation. In this context, it is of paramount importance to introduce novel drug delivery peptides which use mainly a translocation mechanism to enter cells such as viral membranotropic peptides, which have not been yet widely exploited. Moreover, it is particularly intriguing the use of Class I viral fusion peptides as drug delivery vectors, as they are characterized by a structural plasticity which seems to be fundamental for membrane interaction and their hydrophobicity together with the presence of a few charged residues allows both electrostatic and hydrophobic interactions which support a translocation mechanism through the membrane bilayer.

Delivery across cellular membranes involves several mechanisms such as direct transfer through cell surface membrane by lipid membrane fusion or transient permeabilization of the cell membrane; or after endocytosis, transfer across vesicular membranes by lipid disruption, pore formation or fusion. Several of these membrane reorganization steps are involved in the entry of viruses or other intracellular microorganisms, and are also triggered by protein toxins and defense peptides [4,5,12]. Related processes are important in other biological events like the intracellular vesicle budding and fusion or fusion of cells, sperm-egg fusion or the immune response [185].

Two fusion peptides belonging to Class I have been so far exploited as delivery vectors, in particular, the influenza and the HIV fusion peptides [186–188]. The fusion peptide of influenza virus has been used as an endosomal escape device. Its structure changes from random coil to α-helix and can insert itself into the endosomal membrane, mediating the endosomal escape of the virus. This peptide and its derivatives have been used to enhance the endosomal escape of polyplex [189,190] or liposome-encapsulated proteins [191,192].

MPG is a chimeric peptide derived from the fusion peptide of HIV-1 and the nuclear localization sequence of SV40 large T antigen [186,187]. The hydrophobic domain of MPG is critical for insertion into membranes. It interacts only with a few lipids, which limits its association with proteoglycans at the cell surface as well as the risk of uptake through the endosomal pathway. Upon interaction with the phospholipids, it folds from a random to a β structure that temporarily affects cell membrane organization, without any associated leakage or toxicity, facilitating insertion into the membrane and initiation of the translocation process. Although MPG cellular uptake may follow several routes, the major cell translocation mechanism is independent of the endosomal pathway and involves transient membrane disorganization associated with folding into β-structures within the membrane bilayer. Another viral peptide that has been used for the intracellular delivery corresponds to a membrane-perturbing domain derived from the protein gH of Herpes simplex virus type 1 [1,63,64,193–197]. The peptide gH625 interacts with biological membranes, contributing to the merging of the viral envelope and the cellular membrane [97,98]. When gH625 is in the helical conformation, the polar residues concentrate on one face of the helix, giving it an amphiphilic character common to fusion peptides of most fusion glycoproteins of enveloped viruses belonging to class I; moreover, gH625 is very effective on inducing lipid mixing of model membranes. gH625 has been shown to strongly interact and to spontaneously penetrate the lipid-phase and insert into membranes. The peptide-lipid interactions are initiated by the arginine residue located at the *C*-terminus; when the arginine is mutated the fusogenic activity of the peptide is strongly impaired. The hydrophobic domain is also crucial for insertion of the peptide in the membrane and corroborates the notion that hydrophobic interactions between fusion proteins and cell-membrane phospholipids initiate membrane perturbation in the early stages of viral fusion. gH625 cellular uptake is thus associated with its ability to interact with membrane lipids and to form a transient helical structure that temporarily affects membrane organization, thereby facilitating insertion into the membrane and translocation.

Many amyloid diseases are neurodegenerative and terminal and affect large portions of the elder population, thus prompting massive scientific interest for research on inhibition of any step of amyloid formation [198–202]. Several strategies are pursued and there is considerable interest in research on amyloid and on inhibiting cytotoxic effects of amyloid and protein aggregation on the path to amyloid formation. In particular, there are several possible strategies to inhibit the formation of amyloid fibrils. One possibility is the use of small molecules which stabilize the native folded state and can inhibit its unfolding and aggregation [198]. It is also possible to inhibit aggregation by sequestering the monomeric peptide or stabilizing non productive aggregation pathways. The development of peptide-based inhibitors of amyloid formation represents another possibility, and in particular, the use of β-sheet breakers that terminate fibril elongation [203]. Small peptides mimicking the hydrophobic central sequence (residues 16–20) of the amyloid β-peptide were able to modulate the kinetics of aggregation. Moreover, peptidomimetics incorporating β-sheet breaker aminoacids such as *N*-methyl amino acids [204], proline [205] or sugar [200] have proved to be valuable molecules for inhibiting aggregation. Characterizing the species which lead to the fibrils formation, is one of the main challenges in this field and an improved understanding is critical for optimizing the therapeutic action of inhibitors of amyloid formation.

## 6. Conclusions

The exact manner used by peptides to interact with membranes and molecular details of this process are still an area of active research and a matter of extensive debate and controversy. Different peptides utilize different interaction mechanisms or combinations of mechanisms. Furthermore, the mechanisms of interaction can change depending on the conditions of the system, such as pH, temperature and concentration of peptide.

In order to understand the biological processes of peptide-membrane interactions, or to design peptides with tailored functionalities for specific applications, we need to establish, with molecular resolution, the link between the structure and physical characteristics (for example, hydrophobicity distribution or charge) of the peptide and the particular interaction mechanism it induces.

It is clear that a better understanding of peptide-membrane interactions at a molecular level not only is important in the elucidation of various biological processes, but also could be instrumental in designing peptides with tailored functionalities, for example, for antibiotic and drug delivery applications.
